# Neuroprotective Effect of *Abelmoschus manihot* Flower Extracts against the H_2_O_2_-Induced Cytotoxicity, Oxidative Stress and Inflammation in PC12 Cells

**DOI:** 10.3390/bioengineering9100596

**Published:** 2022-10-21

**Authors:** Shih-Wei Wang, Chi-Chang Chang, Chin-Feng Hsuan, Tzu-Hsien Chang, Ya-Ling Chen, Yun-Ya Wang, Teng-Hung Yu, Cheng-Ching Wu, Jer-Yiing Houng

**Affiliations:** 1 School of Medicine, College of Medicine, I-Shou University, Kaohsiung 82445, Taiwan; 2 Division of Allergy, Immunology, and Rheumatology, Department of Internal Medicine, E-Da Hospital, Kaohsiung 82445, Taiwan; 3 School of Medicine for International Students, College of Medicine, I-Shou University, Kaohsiung 82445, Taiwan; 4 Department of Obstetrics & Gynecology, E-Da Hospital/E-Da Dachang Hospital, Kaohsiung 82445, Taiwan; 5 Division of Cardiology, Department of Internal Medicine, E-Da Hospital/E-Da Dachang Hospital/E-Da Cancer Hospital, Kaohsiung 82445, Taiwan; 6 School of Chinese Medicine for Post-Baccalaureate, College of Medicine, I-Shou University, Kaohsiung 82445, Taiwan; 7 Department of Nutrition, I-Shou University, Kaohsiung 82445, Taiwan; 8 Department of Chemical Engineering, I-Shou University, Kaohsiung 82445, Taiwan

**Keywords:** *Abelmoschus manihot*, neurodegenerative disease, oxidative stress, proliferation, inflammation, nucleotide excision repair

## Abstract

The progression of neurodegenerative diseases is associated with oxidative stress and inflammatory responses. *Abelmoschus manihot* L. flower (AMf) has been shown to possess excellent antioxidant and anti-inflammatory activities. This study investigated the protective effect of ethanolic extract (AME), water extract (AMW) and supercritical extract (AMS) of AMf on PC12 neuronal cells under hydrogen peroxide (H_2_O_2_) stimulation. This study also explored the molecular mechanism underlying the protective effect of AME, which was the best among the three extracts. The experimental results showed that even at a concentration of 500 μg/mL, neither AME nor AMW showed toxic effects on PC12 cells, while AMS caused about 10% cell death. AME has the most protective effect on apoptosis of PC12 cells stimulated with 0.5 mM H_2_O_2_. This is evident by the finding when PC12 cells were treated with 500 μg/mL AME; the viability was restored from 58.7% to 80.6% in the Treatment mode (*p* < 0.001) and from 59.1% to 98.1% in the Prevention mode (*p* < 0.001). Under the stimulation of H_2_O_2_, AME significantly up-regulated the expression of antioxidant enzymes, such as catalase, glutathione peroxidase and superoxide dismutase; promoted the production of the intracellular antioxidant; reduced glutathione; and reduced ROS generation in PC12 cells. When the acute inflammation was induced under the H_2_O_2_ stimulation, AME significantly down-regulated the pro-inflammatory cytokines and mediators (e.g., TNF-α, IL-1β, IL-6, COX-2 and iNOS). AME pretreatment could also greatly promote the production of nucleotide excision repair (NER)-related proteins, which were down-regulated by H_2_O_2_. This finding indicates that AME could repair DNA damage caused by oxidative stress. Results from this study demonstrate that AME has the potential to delay the onset and progression of oxidative stress-induced neurodegenerative diseases.

## 1. Introduction

Oxidative stress and inflammation have been implicated in the development of neurodegenerative diseases, including stroke, post-stroke cerebral ischemia–reperfusion, Huntington’s disease, Alzheimer’s disease and Parkinson’s disease [1,2,3]. Oxidative stress leads to excessive production and progressive accumulation of reactive oxygen species (ROS) such as hydrogen peroxide (H_2_O_2_), hydroxy free radicals and superoxide anion, as well as reactive nitrogen species (RNS) such as nitric cation, nitrogen dioxide and peroxynitrite anion, in various pathological conditions. Oxidative stress, an important cause of neuronal degeneration and injury, induces cell apoptosis or necrosis through cellular oxidative damage such as DNA damage, lipid peroxidation, protein oxidation, expression of inflammatory and apoptotic genes and decrease in nitric oxide bioactivity [4,5,6]. Inflammation is known to be essential in regulating the central nervous system. However, this function may run out of control when microglial cells are over-activated and pro-inflammatory cytokines or mediators are over-produced, ultimately leading to neuronal cell damage and dysfunction [3,7].

Hydrogen peroxide is produced from oxidative energy metabolism by the reaction with oxidant-generating agents in vivo. It has the ability to cross lipid bilayers and react with membrane or protein-bound metal ions to form hydroxyl radicals, making H_2_O_2_ an extremely dangerous reactive oxygen [8]. It causes oxidative damage to cells and various lesions in DNA, which causes DNA helixes to twist, hindering base pairing, transcription and replication. Unrepaired oxidative damage can easily cause cancer and shorten telomeres, leading to premature aging and senescence. Mammals have a DNA repair mechanism that can repair individual base damages or genomes by recognizing specific DNA spiral distortions. The nucleotide excision repair (NER) mechanism is the common function that combines with a variety of proteins in a spatially and temporally specific manner to repair major lesions. It repairs bulky DNA adducts and helix-twist damages and is also a synergistic system of base excision repair that can be used to repair oxidative stress-induced DNA damage [9,10].

Many studies have shown that the application of antioxidative and anti-inflammatory strategies to alleviate oxidative stress-stimulated neuronal damage by scavenging free radicals and inhibiting amyloid deposition in neuronal cells is promising in the treatment of neurodegenerative diseases [11,12]. Numerous studies have also reported that some plant extracts or active plant ingredients have a protective effect on neuronal cells and can attenuate the progression of neurological diseases [13,14,15].

*Abelmoschus manihot* (L.) Medic, a member of the Malvaceae family, is widely distributed in valleys and grasslands from Asia to Europe. *A. manihot* flowers (AMf) have been used to treat malignant skin ulcers, burns and cellulitis [16,17,18,19,20,21]. AMf extracts and their bioactive components possess many biochemical activities, including antioxidant, anti-inflammatory, antiviral, anti-diabetic nephropathy, anti-lipogenic, antidepressant, antiplatelet, analgesic, anticonvulsant, cardioprotective, hepatoprotective, immunomodulatory; they are also effective against the cerebral infarction, diarrhea, bone loss, preventing menorrhagia, relieving labor, stimulating lactation and enhancing sexual arousal and reproduction [18,22,23,24]. In China, AMf extracts have been applied in clinical practice for the treatment of diabetic nephropathy and chronic glomerulonephritis [25,26]. In terms of neuroprotective activity, AMf extract has been shown to significantly reduce the incidence of cerebral edema in rats with acute incomplete cerebral ischemia and alleviate the pathological changes in brain tissues [27]. In addition, AMf extract could protect rat hippocampal neurons by inhibiting NMDA (*N*-methyl-D-aspartate) receptor responses [28]. However, the molecular mechanism involved in the neuroprotective activity of AMf extracts remains to be elucidated.

In this study, differentiated rat adrenal pheochromocytoma PC12 neuronal cells, a cell model often used to study neurodegenerative diseases [29,30], were applied to evaluate the neuroprotective effect of AMf extracts on the growth of neuronal cells stimulated by H_2_O_2_, and effects on H_2_O_2_-induced oxidative stress and acute inflammatory response in PC12 cells. The effect of AMf extract on the repair of DNA damage through the NER pathway was also examined.

## 2. Materials and Methods

### 2.1. Preparation of AMf Extracts

The AMf raw materials were purchased from Kangmei Chinese Medicine Store (Bozhou, Anhui, China), and its nucleotide sequence was 99.43% identical to that of GenBank ID: KY218782.1 [24]. In order to prepare AMf ethanol (AME) extract, 2.6 kg of dried flowers were ground to powder and then extracted at room temperature with 16 L of 95% ethanol for 24 h. After filtering and collecting the extract solution, the residue was extracted two more times with 16 L of 95% ethanol each. The extraction solutions were pooled, the solvent was removed using an evaporator (Panchum Scientific, Kaohsiung City, Taiwan), and the residue was dried with a freeze-dryer (Panchum Scientific); finally, the dried AME samples were obtained.

In order to prepare AMf water (AMW) extract, 1.0 kg of dried flower powder was heated in 6 L of water to boiling point and then extracted at 90 °C for 3 h. Then the extract was cooled down to room temperature and filtered. The filtrate was evaporated with an evaporator and finally freeze-dried the residue to obtain the AMW extract

The AMf supercritical-CO_2_ fluid (AMS) extract was prepared by extracting 1.0 kg of flower powder in a supercritical fluid extractor (5 L/1000 bar R&D unit, Natex, Ternitz, Austria). The extraction procedure was as follows: the tank temperature was raised to 40 °C and then kept at this temperature; then, the tank pressure was raised to 150 bar in 20 min, 250 bar in 20 min, 300 bar in 10 min, 350 bar in 10 min and stayed at 350 bar for 2 h. After the extraction, freeze-dried the residue to obtain AMS extract.

These three dried extracts were all kept in a –20 °C freezer until use. The total polyphenol content (TPC) and total flavonoid content (TFC) of the various extracts were analyzed using the method of Tsai et al. [31] and expressed as gallic acid equivalents and catechin equivalents, respectively. The contents of five flavonoids were analyzed by HPLC, according to the method of Chang et al. [24].

### 2.2. Cell Culture and Analyses

The rat adrenal pheochromocytoma PC12 cell line was obtained from Bioresource Collection and Research Center (Hsinchu, Taiwan). The cells were cultivated in Dulbecco’s Modified Eagle Medium supplemented with 10% fetal bovine serum (Gibco, Grand Island, NY, USA), 100 units/mL penicillin—100 μg/mL streptomycin, 1% l-glutamine, 0.02% NaHCO_3_, pH 7.2–7.4. The cells were cultured in an incubator at 37 °C, with an atmosphere of 5% CO_2_ and 95% air.

The general protocol of cell culture in this study was to plate 5 × 10^4^ cells in each well of 96-well plates and incubate for 24 h. In the Treatment mode, PC12 cells were first stimulated with H_2_O_2_ for 4 h and then treated with different concentrations of the extracted sample for 24 h. In the Prevention mode, PC12 cells were first treated with different concentrations of the extracted sample for 24 h and then stimulated by H_2_O_2_ for 4 h.

Cell viability was analyzed using the MTT (3-(4,5-Dimethylthiazol-2-yl)-2,5-diphenyltetrazolium bromide) assay kit (Sigma-Aldrich Chemicals, St. Louis, MO, USA). The medium was removed from the 96-well dish, and after washing the cells with PBS (phosphate buffered saline), 100 μL of 5 mg/mL MTT were added and incubated for 2–4 h. Subsequently, after replacing the MTT reagent with 100 μL of DMSO (dimethyl sulfoxide), the dish was shaken to dissolve the crystals. The amount of PC12 cells was then measured using an ELISA reader at 570 nm wavelength.

Morphological changes in cell nuclei were detected using Hoechst staining. Cells were first fixed with 3.7% paraformaldehyde for 30 min, washed with PBS and stained with Hoechst 33,258 (Sigma-Aldrich) for 30 min at 37 °C. The morphological changes were observed and photographed with a fluorescence microscope.

### 2.3. Assay of Cellular Oxidative System

#### 2.3.1. Intracellular ROS Content of H_2_O_2_-Stimulated PC12 Cells

Intracellular ROS content was analyzed by the Fluorometric Intracellular ROS Kit (Sigma-Aldrich), which detected ROS with 2′,7′-dichlorodihydrofluorescein diacetate (DCF-DA). The cells treated with AME for 24 h and stimulated by H_2_O_2_ for 4 h were washed with PBS and then reacted with 5 μM DCF-DA at room temperature for 30 min. The ROS content was then determined by fluorescence at 502 nm excitation and 524 nm emission in a multi-detection microplate reader (Synergy^TM^ 2, BioTek, Winooski, VT, USA).

#### 2.3.2. Antioxidant Enzymes Activity and Glutathione Content

Cell extracts were prepared from PC12 cells, which were pretreated with AME for 24 h and then stimulated by H_2_O_2_ for 4 h, according to the manufacturer’s instructions. The activities of catalase, glutathione peroxidase (GPx) and superoxide dismutase (SOD) and the content of reduced glutathione (GSH) were analyzed by Sigma-Aldrich assay kits with catalog numbers STA-341, K762-100, STA-340 and STA-312, respectively. The protein content in cell extract was determined by the Bicinchoninic Acid Protein Assay Kit (Sigma-Aldrich).

### 2.4. Western Blot Assay

Western blot was used to detect the protein expression of antioxidant enzymes, inflammatory cytokines, NER-related proteins and β-actin internal standard. This assay mainly followed the procedures described in our previous paper [32]. Briefly, PBS-washed PC12 cells were lysed with a modified RIPA buffer (Sigma-Aldrich). A certain amount of protein was denatured by heating at 95 °C for 5 min. After cooling, it was placed in the sample tank for electrophoresis. The proteins were separated on an SDS-PAGE gel, then transferred to a PVDF membrane, and a primary antibody was added. Subsequently, the separated protein was detected with ECL Plus Western Blotting Detection Reagents (Sigma-Aldrich) after treatment with a secondary antibody conjugated to horseradish peroxidase (HRP). All antibodies used were purchased from Sigma-Aldrich (Table 1). The ChemiDoc XRS+ system (Bio-Rad, Hercules, CA, USA) was used to detect protein expression, and the Quantity One^®^ software (version 5.2.1, Bio-Rad) was used for quantification.

### 2.5. Statistical Analysis

Each experiment was repeated at least three times, and the data were presented as arithmetic means ± standard deviation (SD). For the chemical composition analysis, the significance of differences between mean values was determined by one-way analysis of variance (ANOVA) and Duncan’s multiple range test when the *p* < 0.05. The Student’s *t*-test was used to examine the statistical difference of each parameter in other experiments. These tests were all performed with SPSS 25.0 software (SPSS Inc., Chicago, IL, USA). The significance of the data difference compared with the vehicle group were defined as * *p* < 0.05, ** *p* < 0.01 and *** *p* < 0.001.

## 3. Results

### 3.1. Extraction Yield and Chemical Composition Analysis of AMf Extracts

**Figure 1:**
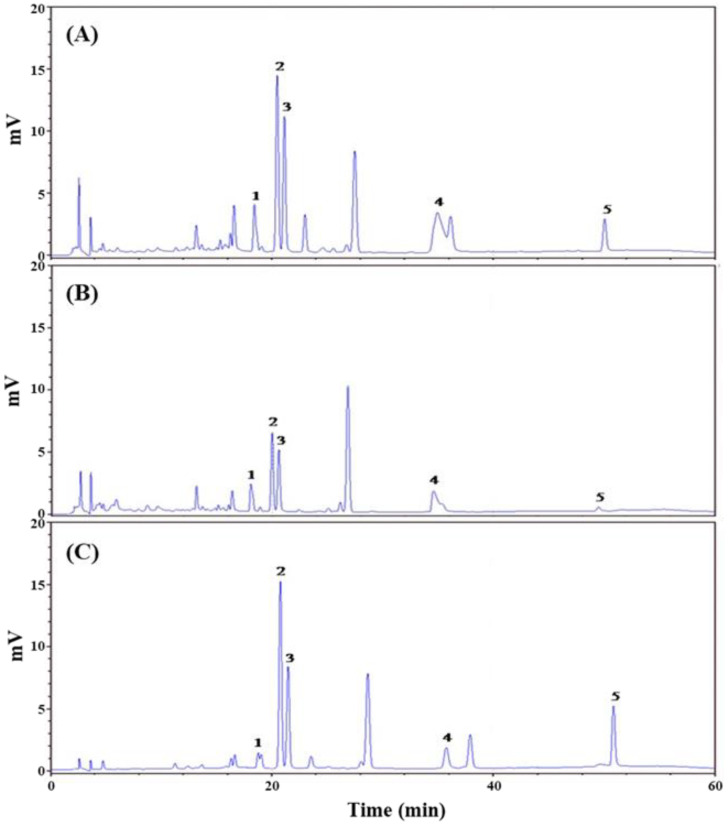
HPLC chromatograms of AMf extracts ((**A**) AME, (**B**) AMW and (**C**) AMS). Identified compounds: rutin (1), hyperoside (2), isoquercitrin (3), myricetin (4) and quercetin (5).

AMf was extracted by 95% ethanol, hot water and supercritical-CO_2_ fluid, and yields were 25.2%, 16.4% and 1.0%, respectively; the extracts were denoted as AME, AMW and AMS, respectively. Previous studies reported that the main active ingredients in AMf extracts are polyphenols and flavonoids [18,33,34]. Therefore, the TPC and TFC in these three extracts were analyzed. Table 2 shows that the richest content of TPC (80.6 mg/g) was found in AME, followed by AMW (61.5 mg/g) and AMS (47.3 mg/g). In terms of TFC, AME was still the highest (38.0 mg/g), followed by AMS (30.9 mg/g) and AMW (28.9 mg/g). The chromatograms of the five major flavonoid ingredients in AMf extracts analyzed by high-performance liquid chromatography (HPLC) are shown in Figure 1, and the content of each ingredient is also shown in Table 2. In these three extracts, hyperoside had the highest content, followed by myricetin, isoquercitrin, rutin and quercetin. Compared with the three extracts, the content of hypericin and quercetin in AMS was higher than that in AME, and the content of other components was still less than that of AME. The content of active ingredients of AMW was generally less than that of AME and AMS.

### 3.2. Cytotoxicity of AMf Extracts on PC12 Cells

**Figure 2:**
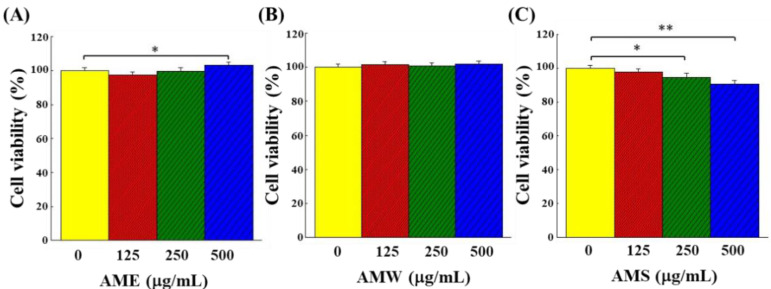
Cytotoxicity of the three AMf extracts on PC12 cells: (**A**) AME, (**B**) AMW and (**C**) AMS. PC12 cells were incubated for 24 h and then treated with specific extract for additional 24 h, and the viability was analyzed with an MTT kit. The vehicle group (group 0) was the PC12 cells grown only with the treatment by the sample solvent DMSO. The cell number of this group was set as 100%. This experiment was repeated five times. The significance of the difference in data compared to the vehicle is labeled as * *p* < 0.05 and ** *p* < 0.01.

The cytotoxicity of the three extracts on PC12 cells was demonstrated by the cell viability. As shown in Figure 2, AME increased cell viability only when concentration reached 500 μg/mL (Figure 2A), AMW did not affect cell viability over the range of concentrations tested (Figure 2B), while AMS exhibited a dose-dependent cytotoxic effect on PC12 (Figure 2C). However, the viability of PC12 cells remained higher than 90% even when AMS concentration was up to 500 μg/mL.

### 3.3. Effects of AMf Extracts on Proliferation of H_2_O_2_-Stimulated PC12 Cells

**Figure 3:**
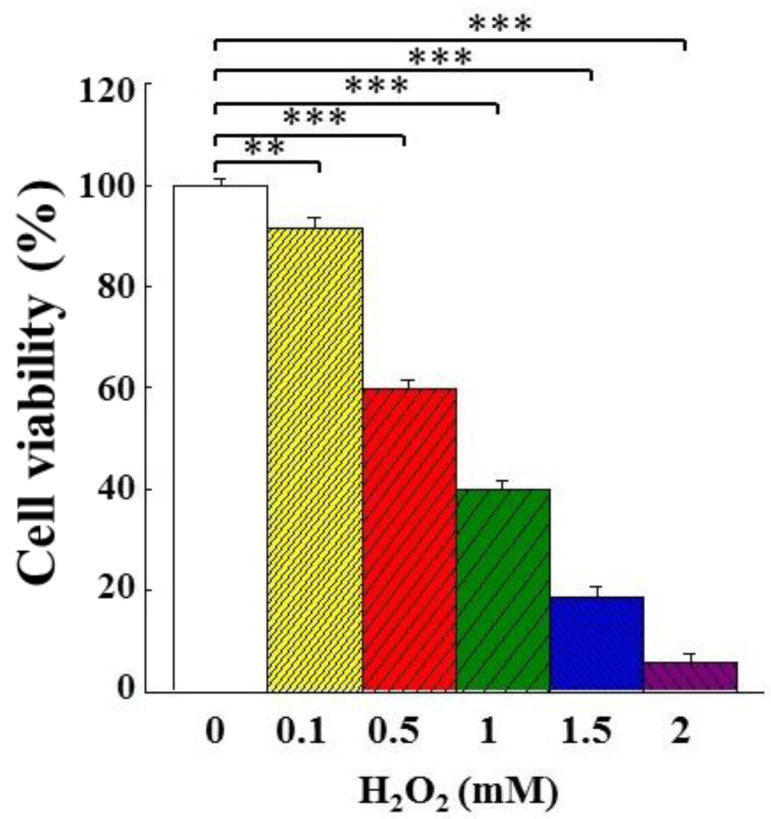
Inhibitory effect of H_2_O_2_ concentration on the growth of PC12 cells. The cells were first incubated for 24 h, then stimulated with the indicated concentration of H_2_O_2_ for 24 h, and cell viability was analyzed with an MTT kit. The vehicle group (group 0) was the PC12 cells grown on the treatment with water. The cell number of this group was set as 100%. This experiment was repeated five times. The significance of the difference in data compared to the vehicle is marked as ** *p* < 0.01 and *** *p* < 0.001.

Cell proliferation (expressed as cell viability) in PC12 cells stimulated with different concentrations of H_2_O_2_ is shown in Figure 3. With the H_2_O_2_ concentration increased, the extent of inhibition on cell proliferation was also increased. When the concentration of H_2_O_2_ was at 0.5 mM, the viability of PC12 cells dropped to 59.9%. In order to observe the protective effect of AMf extracts on the H_2_O_2_-stimulated PC12 cells, the H_2_O_2_ concentration was set at 0.5 mM in the subsequent experiments.

**Figure 4:**
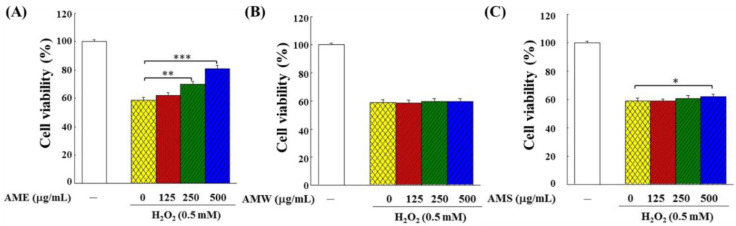
Protective effects of AMf extracts ((**A**) AME, (**B**) AMW and (**C**) AMS) on growth of PC12 cells under the H_2_O_2_ stimulation in Treatment mode. PC12 cells were first cultivated for 24 h, stimulated with 0.5 mM H_2_O_2_ for 4 h, then treated with specific extract for 24 h, and the viability was analyzed with an MTT kit. The control group was the cells grown without H_2_O_2_ stimulation and extract sample treatment, and the cell number of this group was set as 100%. This experiment was repeated five times. The significance of the difference in data compared to the vehicle are labeled as * *p* < 0.05, ** *p* < 0.01 and *** *p* < 0.001.

The protective effects of AMf extract on the growth of the H_2_O_2_-challenged PC12 cells were examined in two modes: (1) in the Treatment mode, the cells were stimulated by H_2_O_2_ for 4 h and then treated with three different extract samples, respectively, for additional 24 h; (2) in the Prevention mode, the cells were treated with three different extract samples, respectively, for 24 h and then stimulated by H_2_O_2_ for another 4 h. Figure 4 shows the experimental results under the Treatment mode operation. AME exhibited the most significant cytoprotective effect, and as the treatment dose increased, the cell viability rose remarkably (Figure 4A). The cytoprotective effect of AMW was not obvious (Figure 4B), while AMS showed a protective effect only when it reached 500 μg/mL (Figure 4C).

**Figure 5:**
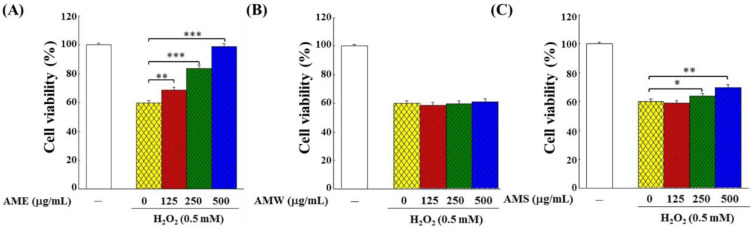
Protective effects of the three AMf extracts on the growth of PC12 cells under the H_2_O_2_ stimulation in Prevention mode: (**A**) AME, (**B**) AMW and (**C**) AMS. The cells were first cultivated for 24 h, treated with specific extract at indicated concentration for another 24 h, then stimulated with 0.5 mM H_2_O_2_ for 4 h. Cell viability was analyzed with an MTT kit. The control group was the PC12 cells grown without treatment with extract sample and H_2_O_2_ stimulation. The cell number of this group was set as 100%. This experiment was repeated five times. The significance of the difference in data compared to the vehicle (group 0) are labeled as * *p* < 0.05, ** *p* < 0.01 and *** *p* < 0.001.

Under the Prevention mode operation, compared to the Treatment mode, AME and AMS had a more profound protective effect on PC12 cells (Figure 5A,C), while AMW had no protective effect (Figure 5B). When 500 μg/mL AME was introduced, the cell viability in the Treatment mode recovered from 58.7% to 80.6%, while that in the Prevention mode recovered from 59.1% to 98.1%. These findings clearly demonstrated that AME had the best protective effect on PC12 cells, and more so in the Prevention mode. Therefore, all subsequent experiments were conducted using AME in the Prevention mode.

### 3.4. Effect of AME Pretreatment on Cell Morphology and Nuclear Staining of H_2_O_2_-Stimulated PC12 Cells

**Figure 6:**
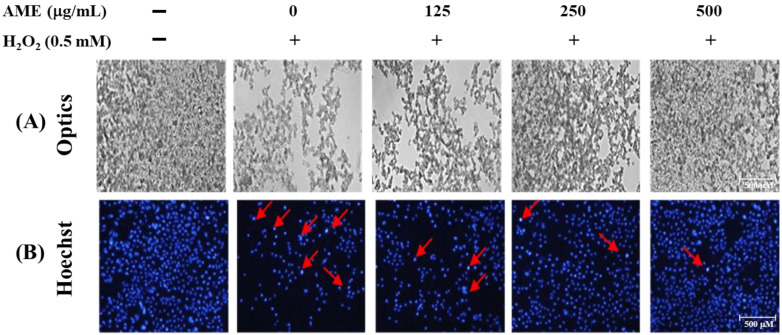
Changes in nuclear morphology of H_2_O_2_-stimulated PC12 cells under the AME pretreatment. The cells were first cultivated for 24 h, treated with AME for another 24 h, then stimulated with 0.5 mM H_2_O_2_ for 4 h. (**A**) The cell morphology was observed by a phase-contrast microscope. (**B**) Nuclear changes were examined by Hoechst staining and observed with a fluorescence microscope. The arrows indicate nuclei with severe DNA damage.

The cell nucleus morphology changes were detected by Hoechst staining and photographed with a fluorescent microscope. Figure 6A shows that the number of PC12 cells significantly decreased when treated with 0.5 mM H_2_O_2._ The cells originally grew in suspension stacking and had become thin and loose after treatment. In Hoechst nucleus staining (Figure 6B), brighter blue fluorescent nuclei marked by the red arrows indicated nuclei with severe DNA damage. When cells were treated with H_2_O_2_, there was a dramatic increase in the number of cells with DNA damage.

When PC12 cells were pretreated with AME, the number of cells increased as the concentration of AME increased; the patterns of cell growth were also returning to a dense and stacked state (Figure 6A). As shown by the Hoechst staining, the number of DNA-damaged cells decreased dramatically when the AME concentration increased (Figure 6B). Therefore, these findings further confirmed that AME possessed a protective effect on the H_2_O_2_-stimulated PC12 cells.

### 3.5. Effect of AME Pretreatment on the Regulation of NER

**Figure 7:**
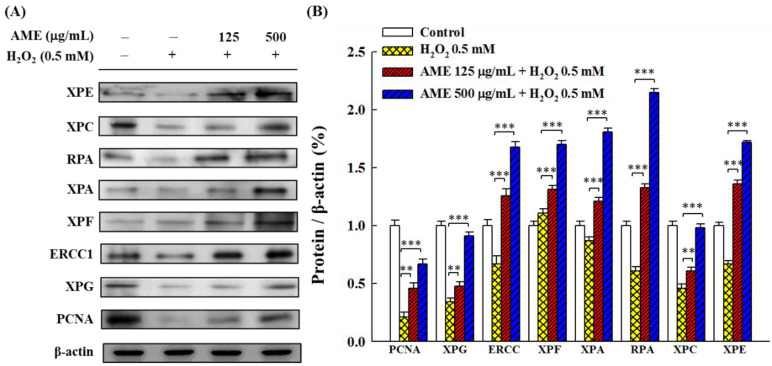
Effect of AME pretreatment on the expressions of NER-related proteins in H_2_O_2_-stimulated PC12 cells. (**A**) Changes in protein expression. (**B**) Densitometric quantitation. The data were estimated from triplicate independent experiments. The control group was the cells grown without treatment with extract sample and H_2_O_2_ stimulation. The protein expressed by this group was set as 1.0. The significance of the difference in data compared to the vehicle group is labeled as ** *p* < 0.01 and *** *p* < 0.001.

NER is a repair mechanism for oxidative stress-induced DNA damage [9,10]. In order to examine whether oxidative stress and AME pretreatment affect the NER system of PC12 cells, this study used Western blot assay to analyze the expression of several NER-related proteins. As shown in Figure 7, when PC12 cells were stimulated with H_2_O_2_ alone, all other proteins were down-regulated (20–86% to the control), with the exception of XPF, which was up-regulated (around 110% to the control). Pretreatment of cells with AME enhanced the production of all tested proteins, implying that AME could substantially raise the NER capacity to repair oxidative stress-induced DNA damage.

### 3.6. Effect of AME Pretreatment on Antioxidant System

**Figure 8:**
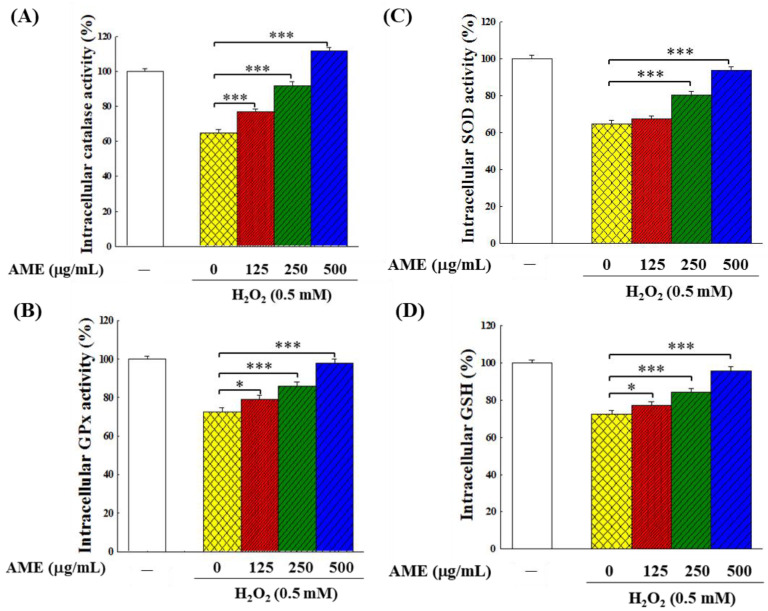
Effect of AME pretreatment on the antioxidant enzymes’ activities and the GSH content in H_2_O_2_-stimulated PC12 cells: (**A**) catalase, (**B**) GPx, (**C**) SOD and (**D**) GSH. The cells were first cultivated for 24 h, treated with AME at the indicated concentration for another 24 h, then stimulated with 0.5 mM H_2_O_2_ for 4 h. The activities of catalase, GPx, SOD and the content of GSH were analyzed. This experiment was repeated five times. The significance of the difference in data compared to the vehicle (group 0) are labeled as * *p* < 0.05 and *** *p* < 0.001.

An increase in the amount of ROS would modulate the intracellular antioxidant system. In this study, the levels of antioxidant enzymes and reduced glutathione (GSH) were used to reflect the changes in the intracellular antioxidant system. Figure 8 indicates that the H_2_O_2_-stimulation significantly decreased (about 64–73% of the control group) the activities of the three intracellular antioxidant enzymes, i.e., catalase, glutathione peroxidase (GPx) and superoxide dismutase (SOD). The H_2_O_2_ stimulation also significantly reduced the GSH content (73.5% of the control group). This finding implies that the H_2_O_2_ stimulation reduced the performance of the antioxidant system in PC12 cells. When cells were pretreated with AME for 24 h and then challenged by 0.5 mM H_2_O_2_, the activities of these three suppressed intracellular antioxidant enzymes and GSH generation were all recovered. The higher the AME concentration, the higher the recovery of these enzymes in cells. At 500 μg/mL, all these four indexes returned (GPx, SOD and GSH) to or exceeded (catalase) those in the control, indicating that AME could protect the intracellular antioxidant system when PC12 cells were subjected to oxidative stress.

**Figure 9:**
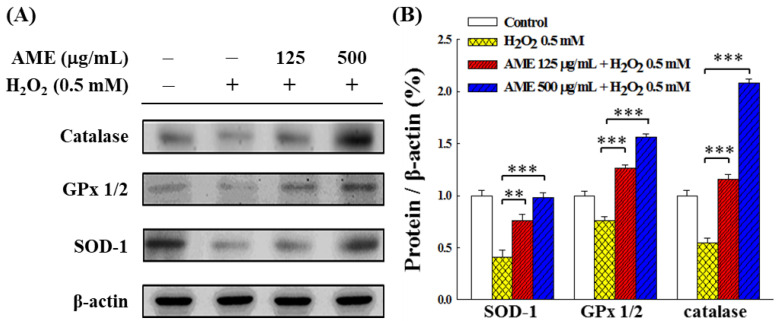
Effect of AME pretreatment on the production of antioxidant enzymes in H_2_O_2_-stimulated PC12 cells. (**A**) Changes in protein formation. (**B**) Densitometric quantitation. The data were estimated from triplicate independent experiments. The control group was the cells grown without treatment with extract sample and H_2_O_2_ stimulation. The protein expressed by this group was set as 1.0. The significance of the difference in data compared to the vehicle group is labeled as ** *p* < 0.01 and *** *p* < 0.001.

In order to examine whether the increase in activity of these antioxidant enzymes was due to increased production of enzyme proteins in the cells, Western blot analysis was carried out. Figure 9 shows that when cells were treated with H_2_O_2_ only, the production of these antioxidant enzymes was significantly inhibited. However, when cells were pretreated with AME, the H_2_O_2_-induced inhibitory effect was suppressed, and the production of all three enzyme proteins was up-regulated in a dose-dependent fashion. When the AME concentration reached 500 μg/mL, the production of these enzyme proteins was comparable to the group (SOD) or exceeded (GPx and catalase) that of the control. This result is in good agreement with the data shown in Figure 8; it shows that the protective effect of AME on the intracellular antioxidant system was achieved by up-regulating the production of antioxidant enzymes.

**Figure 10:**
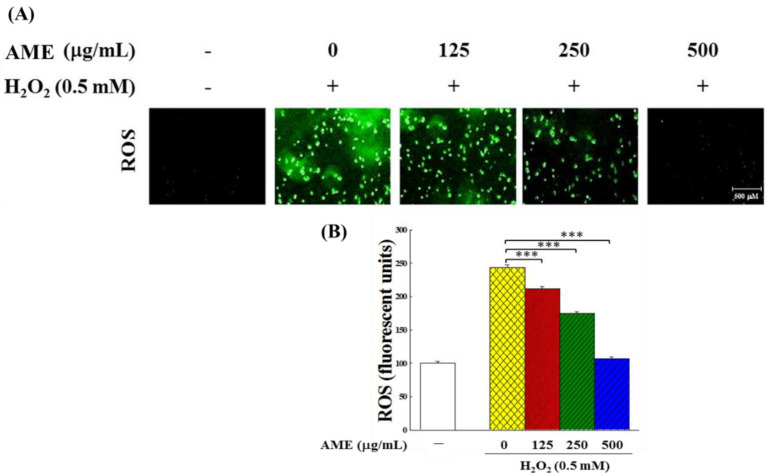
Effect of AME pretreatment on the ROS generation in H_2_O_2_-stimulated PC12 cells. (**A**) The intracellular ROS levels indicated by DCF-DA fluorescence intensity. (**B**) Fluorescent quantitation. This experiment was repeated three times. The significance of the difference in data compared to the vehicle (group 0) is labeled as *** *p* < 0.001.

When PC12 cells were challenged with 0.5 mM H_2_O_2_, the intracellular ROS production, detected by fluorescence microscope, increased significantly by 1.43 times compared with the control group (Figure 10A,B). However, when PC12 cells were pretreated with AME and then stimulated with H_2_O_2_, the amount of intracellular ROS decreased in direct relationship with the rise of AME concentration. As the AME concentration reached 500 μg/mL, the intracellular ROS content dropped down to the same level as the control group (no H_2_O_2_ induction).

### 3.7. Effect of AME Pretreatment on Inflammatory Responses

**Figure 11:**
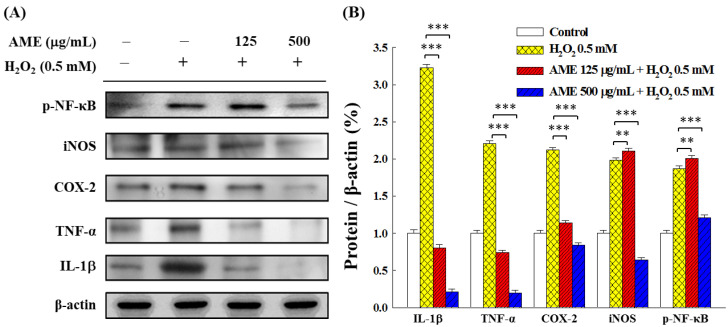
Effect of AME pretreatment on the expressions of pro-inflammatory cytokines and mediators in H_2_O_2_-stimulated PC12 cells. (**A**) Changes in protein expression. (**B**) Densitometric quantitation. The data were estimated from triplicate independent experiments. The control group was the PC12 cells grown without treatment with extract sample and H_2_O_2_ stimulation. The protein expressed by this group was set as 1.0. The significance of the difference in data compared to the vehicle group is labeled as ** *p* < 0.01 and *** *p* < 0.001.

Oxidative stress can stimulate the inflammatory response. Figure 11 shows that the H_2_O_2_ induction substantially increased the expression of pro-inflammatory cytokines and mediators (e.g., IL-1β, TNF-α, COX-2 and iNOS) and phosphorylation of NF-κB in PC12 cells. Pretreatment with AME, even at a low AME dose (125 μg/mL), significantly reduced the expression of IL-1β, TNF-α and COX-2. However, effectively down-regulating the iNOS expression and NF-κB phosphorylation required AME concentration at 500 μg/mL, indicating that AME pretreatment was less sensitive in regulating the iNOS expression and NF-κB phosphorylation.

## 4. Discussion

The present study investigated the protective effects of three AMf extracts on the growth of PC12 neuronal cells stimulated by H_2_O_2_. This study also examined the inhibitory effects of AME pretreatment on intracellular ROS production and acute inflammatory response and enhancing effects on intracellular antioxidant system and NER capacity. It should be noted here that the immortalized PC12 cell line is a model of neuronal cells but does not fully represent neurons.

To date, there are more than 128 chemical constituents identified mostly from the flowers but also from seeds, stems and leaves of *A. manihot*. These components can be classified as polyphenols, flavonoids, amino acids, nucleosides, steroids, organic acids, volatile oils and polysaccharides [18]. Polyphenols and flavonoids are recognized and widely studied as the main active substances of AMf [20,33,34]. Among them, hyperoside, myricetin, isoquercetin, rutin, quercetin, hibifolin and quercetin-3-O-robinobioside are regarded as the main active ingredients [33,34]. In this study, the content of five flavonoids in three extracts of AMf was analyzed by HPLC, as shown in Figure 1 and Table 2. Among the three extracts, AME has the highest TPC and TFC values. In terms of active ingredient content, only AMS has a higher content of hypericin and quercetin than AME, while the content of other components of AMS and all components of AMW was lower than that of AME.

Polyphenols and flavonoids can scavenge reactive oxygen species and nitrogen species and activate redox transcription factors to regulate gene expression, so they often possess antioxidant, anti-inflammatory, anticancer, antiviral and neurological and cardiac protection activities. Thus, these cost-effective medicinal ingredients have significant biological activity. Depending on the type, mode of action and bioavailability, polyphenols and flavonoids have been proven to be effective in managing a variety of diseases [35,36,37].

When the extraction is carried out with different solvents, types of ingredients can be extracted depending on the characteristics of the solvent. In this study, hot water, 95% ethanol and supercritical CO_2_, the polarity ranks from high to low, were used to prepare AMW, AME and AMS. By comparing the extraction yield and the TPC and TFC in the extract (Table 2), it can be seen that ethanol had the highest extraction efficiency. The results from this study have also shown that AME exhibited the best protective effect on the H_2_O_2_-stimulated PC12 cells, which may be related to the high content of TPC and TFC in this extract.

Incubation with AME and AMW did not affect the growth of PC12 cells, but AMS showed slight cytotoxicity (Figure 2). AME exhibited a significant protective effect on the proliferation of the H_2_O_2_-induced PC12 cells, while AMS had little effect, and AMW had no effect (Figure 4 and Figure 5). This shows that the components contained in the three extracts should be different, and it is worth further analysis of the differences in the components contained in these three extracts.

Oxidative stress induced by H_2_O_2_ stimulation generally leads to apoptosis of most human cells [38,39]. Previously, numerous studies reported that AMf extracts could inhibit oxidative stress-induced apoptosis through regulation of caspase-3/8/9, Bax and Bcl-2 in cells such as glomerular podocyte [25,40], human umbilical vein endothelial cells (HUVECs) [41], rat kidney epithelial NRK-52E cells [42] and human hepatoma HepG2 cells [43]. This information prompted us to examine whether AME could also protect the growth of PC12 cells by inhibiting apoptosis. Furthermore, oxidative stress can lead to DNA damage. The Hoechst staining experiment shown in Figure 6B demonstrated that AME had the effect of protecting intracellular DNA.

The NER mechanism, a common DNA repair system, can recognize lesions and bind to a variety of proteins in a spatially and temporally specific manner to remove damaged, short single-stranded DNA fragments and synthesize short complementary double strands using undamaged single-stranded DNA as a template, to repair oxidative stress-induced DNA damage [10,44,45].

The NER involves two major sub-pathways: global genomic NER (GG-NER) and transcription-coupled NER (TC-NER). These two sub-pathways differ in how they recognize DNA lesions but function similarly in cleavage, repair and ligation of damaged genes. The former is responsible for repairing damages throughout the genome, while the latter is responsible for lesions in active gene transcription [46].

NER in mammalian cells involves nine main proteins. The deficiency of specific proteins can lead to specific diseases; for example, XPA, XPB, XPC, XPD, XPE, XPF and XPG are derived from xeroderma pigmentosum and belong to the GG-NER sub-pathway, while CSA and CSB proteins are associated with Cockayne syndrome, which belongs to the TC-NER sub-pathway. In addition, ERCC1, RPA and PCNA are involved in the excision and repair of nucleotides.

It was reported that H_2_O_2_ stimulation reduces the expression of most related proteins in the NER system [9,47]. This study illustrated that the challenge of the H_2_O_2_-induced oxidative stress reduced the expression of most NER-related proteins tested to 20–86% while slightly increasing the expression of XPF to 110% (Figure 7). Both XPF and XPG are endonucleases that specifically incise damaged strands at 50 and 30 from lesions, respectively, and facilitate the release of lesions containing 22–32 nt long oligomers [10]. ERCC1 expression is considered to be a major key target for redox-regulated NER capabilities [47]. It was shown that the incision activity of ERCC1 requires heterodimerization with XPF (ERCC4) [48]; however, the expression of ERCC1 and XPF are not correlated [47,49]. This might explain why H_2_O_2_ significantly retarded the expression of XPG, whereas it enhanced the formation of XPF in PC12 cells, as shown in this study. Further studies are needed to clarify this difference. Nonetheless, the present study found that treatment with AME up-regulated all tested NER-related proteins in the H_2_O_2_-stimulated cells, showing that AME could protect the NER system to maintain the DNA repair function in cells under oxidative stress.

The results in this study show that AME pretreatment could effectively decrease intracellular ROS, enhance GSH content and up-regulate the production of antioxidant enzymes in the H_2_O_2_-stimulated PC12 cells (Figure 8, Figure 9 and Figure 10). AME could also attenuate the inflammatory responses which were induced by H_2_O_2_ stimulation (Figure 11). Our findings are consistent with those reported in the literature. Deng et al. reported that Huangkui capsule, a flavonoid extract of AMf, could suppress inflammation and oxidative stress in the lipopolysaccharide-induced RAW 264.7 cells and mice [50]. Huangkui capsule could also attenuate renal fibrosis in diabetic nephropathic rats by modulating oxidative stress [51]. Liu et al. reported that ethyl acetate extract of AMf could exert significant suppressive effects on oxidative stress in the H_2_O_2_-stimulated HepG2 cells and D-galactose-stimulated aging mice [43]. The histological analysis demonstrated that this extract effectively reduced the brain and liver injury of these mice. Qiu et al. also reported similar results [52]. Moreover, AMf extract was shown to protect mice against CCl_4_-induced liver injury through anti-oxidative stress and anti-inflammatory effects [53]. AMf extract also exhibited a hepatoprotective effect on the α-naphthyl isothiocyanate-induced cholestatic liver damage through anti-inflammation, anti-oxidative injury and regulation of liver transporters expression [54].

## 5. Conclusions

This study demonstrates that the administration of AME can protect the survival of cells, upregulate the expression of intracellular antioxidant enzymes, promote the generation of intracellular GSH and substantially decrease ROS in the H_2_O_2_-stimulated PC12 neuronal cells. In addition, AME has significant anti-inflammatory activity on the cells and the repairing effect on the NER system to reduce gene damage caused by oxidative stress. Therefore, AME has the potential to be useful in the prevention and treatment of neurological diseases caused by oxidative stress and inflammation.

## Figures and Tables

**Table 1:** The sources of the antibodies used in this study.

1^∘^ Ab	2^∘^ Ab	Molecular Weight (kDa)
Catalase	Rabbit	60
GPx 1/2	Rabbit	24
SOD-1	Mouse	16
*p*-NF-κB	Rabbit	65
iNOS	Rabbit	130
COX-2	Rabbit	75
TNF-α	Rabbit	25
IL-1β	Rabbit	17
XPE	Rabbit	127
XPC	Rabbit	106
RPA	Rabbit	37
XPA	Rabbit	31
XPF	Rabbit	104
ERCC	Rabbit	36
XPG	Rabbit	133
PCNA	Rabbit	36
β-actin	Mouse	48

**Table 2:** Extraction yield and chemical composition of the three AMf extracts *.

Extract	AME	AMW	AMS
Extraction yield (*w*/*w* %)	25.2	16.4	1.0
TPC (mg/g extract)	120.8 ± 0.8 ^A^	61.5 ± 1.4 ^B^	47.3 ± 0.8 ^C^
TFC (mg/g extract)	57.0 ± 1.0 ^A^	28.9 ± 1.7 ^B^	30.9 ± 1.4 ^B^
Rutin (1)	10.0 ± 0.4 ^Ac^	6.8 ± 0.2 ^Bc^	5.0 ± 0.2 ^Cc^
Hyperoside (2)	37.8 ± 2.1 ^Ba^	18.9 ± 1.2 ^Ca^	42.4 ± 2.2 ^Aa^
Isoquercitrin (3)	20.6 ± 1.7 ^Ab^	10.9 ± 1.3 ^Bb^	17.9 ± 1.0 ^Ab^
Myricetin (4)	36.9 ± 1.4 ^Aa^	17.5 ± 0.5 ^Ba^	19.2 ± 1.1 ^Bb^
Quercetin (5)	2.6 ± 0.1 ^Bd^	1.1 ± 0.1 ^Cd^	4.1 ± 0.3 ^Ac^

* The significance of differences between mean values was determined by one-way ANOVA and Duncan’s multiple range test when the *p* < 0.05. ^A, B, C^ Different letters in the same row indicate significant differences (*p* < 0.05). ^a, b, c^ Different letters in the same column indicate significant differences (*p* < 0.05).

## Data Availability

Data are contained within the article.
